# Analysis of 14 BAC sequences from the *Aedes aegypti *genome: a benchmark for genome annotation and assembly

**DOI:** 10.1186/gb-2007-8-5-r88

**Published:** 2007-05-22

**Authors:** Neil F Lobo, Kathy S Campbell, Daniel Thaner, Becky deBruyn, Hean Koo, William M Gelbart, Brendan J Loftus, David W Severson, Frank H Collins

**Affiliations:** 1 Center for Global Health and Infectious Diseases, Department of Biological Sciences, University of Notre Dame, Notre Dame, IN 46556-0369, USA; 2 Harvard University, Cambridge, MA 02138, USA; 3 TIGR, Rockville, MD, 20850, USA

## Abstract

**Background:**

*Aedes aegypti *is the principal vector of yellow fever and dengue viruses throughout the tropical world. To provide a set of manually curated and annotated sequences from the *Ae. aegypti *genome, 14 mapped bacterial artificial chromosome (BAC) clones encompassing 1.57 Mb were sequenced, assembled and manually annotated using a combination of computational gene-finding, expressed sequence tag (EST) matches and comparative protein homology. PCR and sequencing were used to experimentally confirm expression and sequence of a subset of these transcripts.

**Results:**

Of the 51 manual annotations, 50 and 43 demonstrated a high level of similarity to *Anopheles gambiae *and *Drosophila melanogaster *genes, respectively. Ten of the 12 BAC sequences with more than one annotated gene exhibited synteny with the *A. gambiae *genome. Putative transcripts from eight BAC clones were found in multiple copies (two copies in most cases) in the *Aedes *genome assembly, which point to the probable presence of haplotype polymorphisms and/or misassemblies.

**Conclusion:**

This study not only provides a benchmark set of manually annotated transcripts for this genome that can be used to assess the quality of the auto-annotation pipeline and the assembly, but it also looks at the effect of a high repeat content on the genome assembly and annotation pipeline.

## Abstract 2

In order to provide a set of manually curated and annotated sequences from the *Aedes aegypti *genome, mapped BAC clones encompassing 1.57 Mb were sequenced, assembled and manually annotated using computational gene-finding, EST matches as well as comparative protein homology.

## Background

*Ae. aegypti *is the primary vector for both dengue and yellow fever viruses. In an effort to better understand this important disease vector and to provide tools to facilitate new avenues of research, whole-genome sequencing has been initiated. The 1.3 Gb genome (strain LVP^ib12^) has been sequenced to 8× coverage in a joint effort by the Broad Institute [1] and The Institute for Genomic Research (TIGR) [2]. The trace reads were assembled with the ARACHNE genome-assembly package [3] into 4,758 supercontigs (assembly *AaegL1*). A collaborative annotation of the genome by VectorBase and TIGR has resulted in Genebuild 1.0 (designated *AaegL1.1*) consisting of 15,419 transcripts [4].

In this era of whole-genome sequencing, assembly and annotation, only a single animal genome - *Caenorhabditis elegans *- has been completely sequenced, resulting in five fully contiguous telomere-to-telomere chromosomal sequences with more than 90% of annotations supported by experimental evidence [5]. This unusually complete animal genome provides a solid set of data for the scientific community. At present, large genomes are usually sequenced as draft versions, resulting in the automatic production of an assembled genome. These consist of sets of contigs (contiguous sequence) that are oriented and ordered (when possible) across gaps with the sequences from the ends of cloned DNA (mate pair information) into supercontigs or scaffolds. These scaffolds are the basis of various analyses such as gene annotation and physical mapping.

Genome assembly can be complicated by the presence of haplotype polymorphisms present in the strain used for genome sequencing, high repeat content, cloning biases, and regions that are duplicated in the genome. The genome of *D. melanogaster *[6] and *A. gambiae *[7] have been through several rounds of assembly and gene annotation, which have each successively resulted in a better and more complete version of the genome consisting of mapped sequence with fewer gaps and an improved set of gene models [6-8].

The quality of a genome annotation depends on factors such as the gene prediction algorithm, the presence of high-quality comparative data such as expressed sequence tags (ESTs) and experimentally validated gene models, and effective masking of repeat and transposon open reading frames (ORFs). The dataset of gene models used to 'train' the algorithm to the specific genome is particularly important. Currently, the highest-quality gene models are those made by expert curators who manually examine all sources of evidence to make a gene prediction (such as that done with model-organism genomes like that of *Drosophila*).

In an effort to provide manually curated regions of the *Ae. aegypti *genome that can be used to assess the automatic annotation of the *Aedes *genome, we have sequenced, assembled and analyzed 14 bacterial artificial chromosome (BAC) clones. This study provides a set of high-quality manually annotated *Aedes *transcripts that have been compared to the other sequenced dipteran genomes - *A. gambiae *and *D. melanogaster*. This study also addresses issues such as the high repeat content and the presence of possibly duplicated regions that may have complicated the assembly of the *Aedes *genome.

## Results

### Assembly

Fourteen BAC clones from an *Ae. aegypti *genomic library were isolated using PCR primers specific to single-copy genetic markers [9]. Shotgun sequences from each BAC were assembled into scaffolds using both the TIGR assembler [2] and Seqman [10]. Scaffolds resulting from the different methods of assembly (see Materials and methods) were consistent with the others. Mate-pair inconsistencies were usually from sequences that were in repeat regions of the scaffolds. A small number of single-copy chimeric clones were observed and their elimination, along with other mate-pair inconsistencies, did not change the assembled sequences.

The majority of sequence gaps were filled using primers designed to the unique sequence flanking gaps. Some primers designed to close these gaps did not produce any PCR products and sequencing reactions with these primers using the BAC clones as template terminated at the same region or were unreadable due to polymerase slippage. All remaining gaps in the 14 BACs were flanked by highly repetitive sequence. Assembled BAC sequences were compared with the genome assembly (BLASTN) to see if they assembled in a similar manner. The gaps present in the BAC clone assemblies were either coincident with gaps present in the genome assembly or sequence diverged in the genome assembly when gaps were not present in the same region (as discussed below).

Contigs from each BAC clone were oriented on the basis of end sequences and mate-pairs. Three BACs (BAC4, BAC7 and BAC8) were each assembled into continuous sequences with no gaps. The remaining BAC sequences assembled into sets of oriented scaffolds with gaps (arbitrarily replaced by 100 Ns) (Table 1). The only BAC clones that showed differences with the assembly made at TIGR were BAC8 and BAC9. Assembled contigs seem to have been mixed during their assembly and a careful assembly (using Seqman) separated the two BAC clones into their respective scaffolds. This was verified with PCR spanning gaps and comparison to the genome assembly. The 14 BAC assemblies totaled 1,571,625 bp (approx 0.12% of the 1.3 Gb genome). The average G+C content of all scaffolds was 37.75%. Although all the sequences had a G+C around the average, BAC3 had the lowest at 27% and BAC2 had the highest at 47% (see Table 1).

**Table 1:** Summary of BAC assemblies

BAC number	Name of BAC	Chromosome arm	GenBank accession number	Genetic marker	Scaffolds in assembly	Total number of contigs	Length (bp)	G+C%
1	ND13I3	2q	EF173370	Rpl17A	1	2	82203	38.97
2	ND22N19	2q	EF173371	D6L600	1	2	146563	47.09
3	ND22N5	3p	EF173372	Mal1	1	2	116923	27.39
4	ND41B18	3p	EF173373	LF347	1	1	164547	37.94
5	ND41C6	2q	EF173374	VMP-15a3	1	7	89409	38.93
6	ND46O19	2q	EF173375	BA67	1	7	114988	38.19
7	ND48J19	2q	EF173376	D7	1	1	83496	36.99
8	ND56P6	3q	EF173377	Para	1	1	81099	37.14
9	ND67B23	3q	EF173378	LF106	2	2	136645	39.29
10	ND83P15	3p	EF173379	AEGI28	1	2	76584	35.15
11	105H24	1p	EF173366	LF178	1	2	140290	38.43
12	124C17	2q	EF173367	LF138	1	8	158121	38.25
13	26O21	2p	EF173368	LF342	1	2	87550	37.00
14	92LO9	3p	EF173369	LF253	1	3	93207	37.72

The 14 BAC clones were localized to chromosome arms with single-locus genetic markers previously determined.

### Repeat content

Repeat masking resulted in the masking of approximately 20% of the sequence. As repeat masking here was based on protein homology, the total sequence consisting of transposon sequence is likely to be higher. Manual annotation and similarity searches with *in silico *predictions and EST hits with transcribed transposon sequences increased the repeat/transposon content to approximately 35%. The *Feilai *element [11] was the most common element, comprising approximately 38% of the repeats. Almost all transposons identified were retrotransposons.

### Gene prediction

*In silico *gene prediction was performed initially on the raw assembled scaffolds. A preliminary (BLASTX) analysis of these predicted transcripts (data not shown) demonstrated that there was a significant amount of over-prediction, gene-splitting and incorporation of random and transposon-based ORFs into gene models. Masking of repeat sequences before gene prediction reduced the number of gene models and this dataset was used as evidence for manual annotation.

Gene models predicted by Genscan [12] and FGENESH [13] before repeat masking often included exons derived from transposon ORFs. An *Aedes *gene was often split into two predictions, with the incorporation of unmasked transposon-based and other random ORFs. In addition, the *ab initio *generated sets of gene models (by Genscan and FGENESH) were different. However, some predicted exons did match *Aedes *ESTs. Several hundred ESTs were identified from the *Aedes *database (e < -100) as well as from the *Drosophila *and *Anopheles *datasets (e < -50). A preliminary BLAST analysis of Aedes ESTs (e = 0.0) demonstrated that a large portion of them (around 30%) mapped to transposon ORFs.

### Manual annotation

The 14 BACs were manually annotated in Apollo [14] using various tiers of evidence like ESTs and comparison to other dipteran peptides (see Materials and methods). Transcripts from the *Anopheles *and *Drosophila *genomes were used in conjunction with *Aedes *ESTs to limit the number of exons to those that had similarity to gene models in the other dipteran genomes. Annotations that did not possess similarity to the two dipteran genomes were also analyzed to include ORFs that may be specific to the *Aedes *genome as well as those that may have diverged significantly from their *Anopheles *or *Drosophila *homologs.

There were a total of 51 manual annotations (Table 2) among the 14 BAC sequences, with BAC2 having no annotated transcripts. Fifty of 51 manual annotations were found in the *Ae. aegypti *1.0 Genebuild (*AaegL1.1*) [4] and 41 of these were identical (see Table 2). The remaining varied in several ways including differences in the 3' or 5' exon (seven transcripts), different intron/exon structure (two transcripts) or the annotation was missing in that region of the genome (one transcript). In all cases, the differences in the manually annotated models were based on *Aedes *EST comparisons, comparisons to annotations and ESTs in the *Drosophila *and *Anopheles *genome as well as confirmation by sequencing of PCR amplicons in a few cases. A number of transcripts differed in the length of the 3' or 5 ' UTRs. These differences were usually 10-20 bp long and not considered discordant with the gene build unless they differed by entire exons. All annotations had nucleotide matches in the *Aedes *genome and most had hits to *Aedes *ESTs. The genomic region encompassing BAC11 had two extra transcripts (*AAEL03517 *and *AAEL02535*). A protein comparison revealed that both genome-annotated transcripts were exons from a rhabdovirus nucleocapsid protein. These were not included in the list of manual annotations.

**Table 2:** Summary of manual annotations

BAC number	Transcript number	*Aedes *transcript	Supercontig	Contig	Sequencing of cDNA	Differences in annotation between manual annotation (MA) and gene build	Replicated transcript in *Aedes *assembly			
							
							*Aedes *transcript	Supercontig	Contig	Notes
1	1	AAEL013103	1.789	24718	-	-	AAEL014498	1.1137	29191	-
	2	AAEL013088	1.789	24718	-	-	AAEL014499	1.1137	29191	-
	3	AAEL013092	1.789	24718	-	-	AAEL014500	1.1137	29189	-
	4	AAEL013582	1.875	25903	Identical	Longer 5' in MA	AAEL015005	1.1393	31331	5' end of transcript matched MA
							AAEL013099	1.789	24718	3' end of transcript matched MA
	5*	AAEL015006	1.1393	31331	Identical	-	AAEL013097	1.789	24718	Identical to AAEL013097
							AAEL013583	1.875	25903	Identical to AAEL013583
	6	AAEL013098	1.789	24719	-	Only 3' coding region lines up	AAEL013098	1.789	24719	Only 3' coding region lines up
3	7*	AAEL009524	1.403	16729	-	-	AAEL000392	1.7	624	-
4	8	AAEL008104	1.301	13837	-	-	-	-	-	-
	9	AAEL008110	1.301	13837	Identical	Different intron/exon structure	-	-	-	-
	10	AAEL008114	1.301	13837	Identical	3' longer in MA	-	-	-	-
	11	AAEL008115	1.301	13837	-	-	-	-	-	-
	12	AAEL008103	1.301	13837	Identical	-	-	-	-	-
	13	AAEL008100	1.301	13837	-	-	-	-	-	-
5	14*	AAEL014561	1.1166	29449	Identical	-	Transcript missing	1.216	10858	-
	15	AAEL014559	1.1166	29447	Identical	-	AAEL006682	1.216	10863	-
6	16*	AAEL014711	1.1232	30069	Identical	Different intron/exon structure	AAEL014491	1.1132	29135	-
	17	AAEL014712	1.1232	30070	-	-	AAEL014495	1.1132	29137	-
	18	AAEL014709	1.1232	30070	Identical	-	AAEL014494	1.1132	29136	-
7	19	AAEL006423	1.204	10417	-	-	-	-	-	-
	20	No transcript	1.204	10417	-	Similar to AAEL003685	-	-	-	-
	21*	AAEL006424	1.204	10417	Identical	-	-	-	-	-
	22	AAEL006417	1.204	10417	Identical	-	-	-	-	-
8	23*	AAEL006019	1.186	9724	Identical	-	AAEL008297	1.312	14138	-
9	24*	AAEL010573	1.488	18854	Identical	-	AAEL000068	1.1	48	-
	25	AAEL010594	1.488	18854	Identical	-	AAEL000046	1.1	48	-
	26	AAEL010587	1.488	18855	Identical	-	AAEL000020	1.1	48	-
	27	AAEL010575	1.488	18855	Identical	-	AAEL000076	1.1	48	-
	28	AAEL000054	1.1	48	-	-	AAEL010578	1.488	18855	-
	29	AAEL010595	1.488	18855	-	-	-	-	-	-
10	30	AAEL007897	1.288	13401	Identical	Extra 3' exon in MA	-	-	-	-
	31	AAEL007893	1.288	13401	-	-	-	-	-	-
	32	AAEL007907	1.288	13401	-	-	-	-	-	-
11	33	AAEL002503	1.59	3864	-	-	-	-	-	-
	34	AAEL002532	1.59	3866	Identical	3' exon absent in MA	-	-	-	-
	35	AAEL002534	1.59	3863	-	-	-	-	-	-
	36	AAEL002523	1.59	3864	-	-	-	-	-	-
12	37	AAEL001205	1.25	1764	-	Longer 5' in MA	-	-	-	-
	38	AAEL001215	1.25	1764	Identical	Longer 3' in MA	-	-	-	-
	39	AAEL001215	1.25	1764	Identical	-	-	-	-	-
	40	AAEL001198	1.25	1761	-	-	-	-	-	-
	41	AAEL001201	1.25	1764	-	-	-	-	-	-
	42	AAEL001210	1.25	1764	-	-	-	-	-	-
13	43	AAEL008780	1.348	15270	-	-	AAEL001693	1.39	2713	-
	44	AAEL008781	1.348	15269	-	-	AAEL001703	1.39	2712	-
	45	AAEL008769	1.348	15269	-	-	AAEL001701	1.39	2711	-
	46	AAEL008778	1.348	15269	-	-	AAEL001681	1.39	2711	-
14	47	AAEL005065	1.140	7865	-	-	AAEL005223	1.146	8136	-
	48	AAEL005085	1.140	7866	-	-	AAEL005220	1.146	8136	-
	49	AEL005237	1.146	8136	-	-	AAEL005088	1.140	7865	-
	50	AAEL005229	1.146	8136	-	-	AAEL005059	1.140	7865	-
	51	AAEL005218	1.146	8136	-	-	No gene build annotation	1.140	7865	-

The 51 manually annotated transcripts (Transcript number) from each BAC clone (BAC number) along with their corresponding transcript (Gene build transcript) from the gene build (*AaegL1*) and their location (supercontig, contig) are listed along with cDNA amplicons if sequenced. Transcripts that were replicated in the genome are also listed along with their corresponding gene build transcript, location and differences with the manual annotation (MA) if any. Manual annotations marked with an asterisk indicate single-copy cDNA-derived genetic markers used to isolate the BAC.

To confirm the annotation and expression of a subset of these annotations, primers were designed to all manually annotated transcripts where the prediction lacked necessary evidence. PCR was performed on cDNA obtained from all stages of the mosquito (see Materials and methods). These sequences were utilized to correct or confirm manual annotations when the curator presented multiple possible gene models or splice sites for a particular sequence. All 20 amplicons sequenced were identical to a curated gene model (see Table 2).

### Replicated segments

Eight of the 14 BAC clones had annotations present more than once in the genome assembly. This was unexpected as these BACs were specifically isolated using validated single-locus genetic markers [9]. These replicated transcripts present in *AaeL1.1 *were virtually identical and usually present along with the same flanking transcripts in different supercontigs. To see if intergenic sequence were also replicated, the assembled BAC scaffolds were compared to the *Aedes *genome assembly scaffolds containing the identical transcripts. Though replicated transcripts were virtually identical, intergenic/intron sequences were usually identical on one replicate while they varied slightly on the other. These eight blocks of sequence were present in complete or partially replicated segments in different parts of the *Aedes *genome assembly, with only one replicate possessing identical intergenic sequence and the rest having slightly variable intergenic sequences.

Some replicated blocks were 'hybrids' of the BAC clone and the genomic duplication. This is seen in BAC14, where all five transcripts are found on two supercontigs in the same order and structure. Intergenic sequences from the first two transcripts are identical to that on supercont1.140 while the remaining transcripts have intergenic sequences corresponding to that on supercont1.146. This is also seen with BAC9, where the last transcript and its intergenic sequence are found on one scaffold while the remaining transcripts and their intergenic sequnce correspond to another scaffold - even though all transcripts are found on both scaffolds.

BAC1 was the most complicated with the five transcripts, being found on four supercontigs. All transcripts were seen in supercont1.789 while the remaining usually terminated at the end of a scaffold or had gaps which did not include all transcripts. These three transcripts were also seen with different intergenic sequences on supercont1.1137. The fourth transcript had the 3' end matching up to this scaffold and the 5' end on supercont1.393. The fifth transcript was found on supercont1.1393, whereas a sixth transcript with identical intergenic sequence was not found in the genome, although transcripts matching it but with varying intergenic sequence were found. These replicated regions were usually flanked by highly repetitive DNA and/or gaps or were present at the end of a supercontig.

### Orthology and synteny

When compared with the *Anopheles *and *Drosophila *gene sets (Table 3), 50 and 43 *Aedes *transcript annotations had orthologous transcripts in the *Anopheles *and *Drosophila *gene sets, respectively. The genes from the two other dipteran genomes that were similar to the manual annotations were almost always orthologs of each other (determined by reciprocal BLASTs) [4]. Although most *Aedes *annotations had a one-to-one relationship in the other genomes, some matches were to genes from multigene families. In some cases, the primary BLAST match was much better than the rest and in these cases, an ortholog was postulated. In cases where a number of transcripts matched the manual annotation with similar e-values, orthologs could not be predicted. A single manual annotation did not have any similarity in either genome, and when compared to other dipteran datasets with less stringent parameters it demonstrated similarity to an *Ae. albopictus *salivary protein.

**Table 3:** Orthology and synteny with *Anopheles gambiae *and *Drosophila melanogaster*

*Aedes aegypti*			*Anopheles gambiae*				*Drosophila melanogaster*		
BAC number	Transcript number	*Aedes *transcripts	Ortholog	E-value	Chromosome	Syntenic block	Ortholog	E-value	Syntenic block

1	1	AAEL013103	ENSANGG00000021076	3.8E-133	3R-37D	Yes	CG31938	9.5E-094	--
	2	AAEL013088	ENSANGG00000026626	5.5E-009	3R-37D	Yes	-	-	-
	3	AAEL013092	ENSANGG00000011837	0.0E+0.0	3R-37D	Yes	CG10413	0.0E+0.0	-
	4	AAEL013582	ENSANGG00000023798	1.1E-035	3R-37D	Yes	CG11247	1.1E-128	-
	5	AAEL015006	ENSANGG00000011941	1E-106	3R-37D	Yes	CG3661	2.2E-104	-
	6	AAEL013098	ENSANGG00000002369	0.0E+0.0	3R-37D	Yes	CG7961	0.0E+0.0	-
3	7	AAEL009524	ENSANGG00000015193	7.3E-281	2R-10C	NA	CG8696	7.2E-226	NA
4	8	AAEL008104	1 to many	9.4E-058	GW	-	1 to many	8.7E-040	-
	9	AAEL008110	ENSANGG00000011807	3E-154	2R-12B	Yes	CG4832	2.5E-078	-
	10	AAEL008114	ENSANGG00000009472	4.4E-037	GW	-	CG12752	1.6E-026	-
	11	AAEL008115	ENSANGG00000011761	2.2E-108	2R-12B	Yes	CG11025	3.2E-037	-
	12	AAEL008103	ENSANGG0000012462	7.90E-061	2R-12B	-	CG7808	2.8E-073	-
	13	AAEL008100	ENSANGG00000009215	1.4E-053	2R-8B	-	CG1078	3E-040	-
5	14	AAEL014561	ENSANGG00000022738	3.0E-009	3R-32C	-	-	-	-
	15	AAEL014559	ENSANGG00000010922/ENSANGG00000022179	3.3E-068	3R-35C	-	-	-	-
6	16	AAEL014711	ENSANGG00000005482	0.0E+0.0	3R-29B	Yes	CG8815	0.0E+0.0	-
	17	AAEL014712	ENSANGG00000022484	8.9E-199	3R-29B	Yes	CG15084	8.3E-170	-
	18	AAEL014709	ENSANGG00000007515	0.0E+0.0	3R-29B	Yes	CG15100	0.0E+0.0	-
7	19	AAEL006423	ENSANGG00000020489	1.1E-006	2L-20C	-	CG40120	6.1E-006	-
	20	No transcript	ENSANGG00000011708	3.5E-026	GW	-	CG33803	6.9E-028	-
	21	AAEL006424	ENSANGG00000027449	3.0E-062	3R-30C	Yes	-	-	-
	22	AAEL006417	ENSANGG00000020969	2.6E-056	3R-30C	Yes	-	-	-
8	23	AAEL006019	ENSANGG00000025048	8.0E-073	2L-20C	NA	CG9907	8.9E-174	NA
9	24	AAEL010573	ENSANGG00000015129	3.6E-060	2R-19B	Yes	CG6684	2E-050	-
	25	AAEL010594	ENSANGG00000014580	3.7E-107	2R-19B	Yes	CG32418	2E-032	-
	26	AAEL010587	ENSANGG00000014498	2E-192	2R-19B	Yes	CG9590	1E-083	-
	27	AAEL010575	ENSANGG00000014455	2E-192	2R-19B	Yes	CG11837	3.6E-196	-
	28	AAEL000054	ENSANGG00000002208	0.0E+0.0	2R-12E	-	CG8651	0.0E+0.0	-
	29	AAEL010595	ENSANGG00000010690	0.0E+0.0	2L-26A	-	CG16982	1.7E-240	-
10	30	AAEL007897	ENSANGG0000016631	5.1E-194	3R-29B	Yes	CG14928	6E-164	-
	31	AAEL007893	ENSANGG00000018850	1.3E-175	2R-12C	-	CG31548	2.3E-137	-
	32	AAEL007907	ENSANGG00000015978	1.7E-200	3R-29B	Yes	CG4629	1.3E-144	-
11	33	AAEL002503	ENSANGG00000015084	3.8E-092	X-1B	Yes	CG1989	3E-082	Yes
	34	AAEL002532	ENSANGG00000015036	4.4E-250	X-1B	Yes	CG3707	6.3E-285	-
	35	AAEL002534	ENSANGG00000012432	6.5E-186	X-5C	-	CG17521	1.9E-167	Yes
	36	AAEL002523	ENSANGG00000015081	7.3E-065	X-1B	Yes	CG1660	6.8E-051	-
12	37	AAEL001205	ENSANGP00000016497	0.0E+0.0	3R-33B	-	CG4244	0.0E+0.0	-
	38	AAEL001215	SNAP_00000004435	9.9E-123	3R-33B	Yes	CG4230	2E-055	-
	39	AAEL001215	ENSANGP00000023803	0.0E+0.0	3R-33B	Yes	CG7269	1.2E-299	-
	40	AAEL001198	ENSANGP00000023805	0.0E+0.0	3R-29B	-	CG8451	4E-236	-
	41	AAEL001201	ENSANGP00000016633	9.8E-041	3R-33B	Yes	-	-	-
	42	AAEL001210	ENSANGP00000016606	1.9E-060	3R-33B	Yes	CG8680	2.3E-052	-
13	43	AAEL008780	ENSANGP00000021694	1.9E-079	2L-21C	Yes	CG2071/CG1304	3.1E-058	-
	44	AAEL008781	ENSANGP00000021694	4.6E-071	2L-21C	Yes	CG2071/CG1304	3.5E-048	-
	45	AAEL008769	ENSANGP00000021694	1.9E-088	2L-21C	Yes	CG2071/CG1304	1.1E-064	-
	46	AAEL008778	ENSANGP00000021867	1.6E-103	2L-21C	Yes	-	-	-
14	47	AAEL005065	ENSANGP00000018910	6E-159	2R-16C	Yes	CG6746	3.7E-134	-
	48	AAEL005085	ENSANGP00000018909	6.3E-084	2R-16C	Yes	CG10652	2.5E-078	-
	49	AEL005237	ENSANGP00000018845	4.9E-130	2R-16C	Yes	CG1298	2.4E-095	-
	50	AAEL005229	ENSANGP00000019493	3.7E-122	2R-16C	Yes	CG6746	8.3E-090	-
	51	AAEL005218	ENSANGG00000016433	7.7E-151	2R-16B	Yes	CG10624	1.9E-121	-

Orthology was determined for each transcript from all 14 BACs. The presence of synteny was also determined for orthologous blocks of transcripts when more than one transcript was present on the BAC clone.

To compare gene sizes between the two mosquitoes, the amount of sequence covered by the orthologous genes in *Aedes *and *Anopheles *were compared. Single-exon genes were usually the same size; however, the size of multiexon genes was directly proportionate to the number of introns in *Aedes*. On average, *Aedes *genes were about 3.9 times the size of their *Anopheles *orthologs. Only one *Aedes *BAC sequence demonstrated any degree of synteny with *Drosophila*. BAC11 had two adjacent transcripts that were found to be next to each other in the *Drosophila *genome. Of the 11 BACs with more than one annotated transcript, nine sequences demonstrated synteny with the *Anopheles *genome. Overall, 38 of the 50 transcripts included in these BACs demonstrated synteny in 10 blocks.

For a summary of each BAC clone assembly and analysis please see Additional data file 1.

## Discussion

Fourteen BAC clones encompassing 1.57 Mb were sequenced, assembled and analyzed for repeat and gene content. Manual gene annotations were compared to the *Ae. aegypti*, *A. gambiae *and *D. melanogaster *gene sets. A subset of these annotations had their expression and sequence confirmed with reverse transcription-PCR (RT-PCR) and sequencing. This benchmark analysis of the *Aedes *genome has yielded a set of manually annotated transcripts that has been validated with molecular and comparative data. In addition, we have presented data that may clarify the origin of duplicated transcripts in the genome assembly.

### BAC assembly

The quality of these BAC assemblies is critical for a valid assessment of the genome assembly and the automatic gene-annotation pipeline. To enable this assessment, each BAC clone was individually assembled using two assembly algorithms and the resulting duplicated assemblies were compared to make sure that contigs were identical. In addition, all BAC sequences were assembled together to ensure that they sorted independently into the contigs corresponding to individual BAC clones. These stringent assemblies revealed that the sequence of BAC9 (GenBank: AC149799), which was submitted to GenBank before this analysis, had contigs in it that were from BAC8 (GenBank: AC149798). A stringent analysis of these BACs in particular enabled their correct assembly. It was interesting to note that gaps present in the final BAC scaffolds were identical to those present in the genome assembly. We believe that the high repeat content of the sequence in the remaining gaps produces tertiary structures that are not conducive to sequencing. A high G+C content may also contribute to this phenomenon. As a result, we were unable to close several gaps. The 14 final assemblies were confirmed both with PCR, sequencing and a comparison to the genome assembly.

### Repeat content

Assembled and oriented BAC scaffolds were masked for repeat sequence to characterize the transposon content as well as to enable a more efficient *in silico *gene model prediction. Gene-prediction algorithms cannot distinguish transposon ORFs, resulting in their being annotated along with species-specific ORFs. Resulting gene models may not be indicative of real genes, as genes could be split, merged or have extra exons. Initial repeat identification demonstrated that the *Aedes *genome has an unusually high repeat content [15]. Repeat masking [16,17] was performed using multiple repeat datasets to maximize the number of repeats identified. An initial analysis of *in silico *gene annotations derived from the masked sequences revealed that a number of transposons were not identified as a result of the incomplete cataloging of the *Aedes *transposon dataset. This is seen with BAC2, where there were no transcripts annotated on the assembled sequence but gene prediction on repeat-masked sequence suggested the presence of up to 18 transcripts that are derived from unmasked transposon ORFs. The high repeat content of this genome is particularly interesting and impacted on the sequencing, assembly, *in silico *and manual annotation presented in this study. The proper identification of a genome's repeat content is vital as it impacts on these analyses that form the basis of genomic studies.

### Manual annotation and RT-PCR

Manually curated genes are generally considered to be the highest tier of gene models for genome annotation and training datasets. Annotations were based on several sets of data that include manual inspection of species-specific ESTs and comparative data. A portion of the ESTs mapped to transposons, complicating the manual annotation. These transposon-related ESTs can be attributed either to active transposition or to genome-related transposition silencing. As a result, *in silico *gene prediction on unmasked sequence resulted in a higher number of predicted genes (around 4 times more), while the presence of unidentified repeat sequences on masked sequence resulted in over-prediction as well. Although most of the ORFs from the 51 final manually annotated gene models were present in these predictions, transposons present in intergenic sequences led to the splitting and merging of exons along with transposon ORFs. Though the resulting gene predictions from the two *ab initio *gene-prediction programs were not alike, they did capture similar exons. These *in silico *predicted exons were helpful in determining splice sites, along with EST and comparative evidence during manual annotation. The large repeat content in this genome highlights the importance of proper repeat identification and masking before gene prediction in annotation pipelines.

Gene models (see Table 2) were predicted only if they had supporting EST and comparative evidence and did not overlap with sequence that was homologous to transposons. We do not believe we have eliminated any 'domesticated' transposons, although this remains a possibility.

PCR performed on a cDNA library confirmed expression of a subset of transcripts, enabled a sequence comparison of the expressed transcripts with the manual annotations and also introduced an annotation quality-control step. To enable the most thorough expression analysis, the cDNA library was derived from RNA extracted from all stages of mosquito development (see Materials and methods). This molecular verification points to the importance of manual annotations in a genome-annotation pipeline that can not only verify the quality of the auto-annotation but also provide a set of high-quality transcripts that can be used to develop and improve it.

### Comparison of gene models to the *Aedes *gene build

All manual annotations were compared to the *Aedes *genome assembly and Genebuild - *AaegL1.1 *(see Table 2). Almost all manually annotated transcripts were found in the *Aedes *gene build. Differences between the manually annotated models and the transcripts from the gene build included a transcript missing, extra transcripts in the gene build and differences in annotation (see Table 3). When looking at nucleotide similarity (BLASTN), only one transcript on BAC7 (number 20, see Tables 2, 3) did not have a match in the gene build, even though it had perfect nucleotide match in the genome. This annotation belonged to a multigene family (histone H3) and had several almost identical annotated transcripts elsewhere in the *Aedes *genome. The sequence flanking this gene model consisted of transposon sequence, and the entire region was labeled as repetitive in the genome assembly [4]. This transcript, present in multiple copies in the genome as well as being flanked by transposon sequence, was masked before mapping of ESTs to the assembled genome and consequent gene annotation. This points to the importance of differentiating multicopy gene sequences versus those that are homologous to transposons and to the necessity of a comprehensive catalog of the *Aedes *transposon dataset.

This set of manually annotated transcripts enables a quality check of the *Aedes *genome auto-annotation. Approximately 12% of the manually annotated transcripts possessed minor differences from their auto-annotation counterparts, indicating a high-quality genome annotation effort. These differences, as well as the identification of a rhabdovirus nucleocapsid incorporation, highlights the importance of manual annotation and points to a few issues an auto-annotation pipeline may have.

### Replicated BAC transcripts in genome assembly

The 14 BACs were identified from single-locus genetic markers [9]. However, eight of these blocks of genomic sequence possessed transcripts (including the single-copy markers) that were replicated in the genome assembly, along with flanking transcripts, in the same order and structure (see Table 2). A further analysis of the single-copy genetic markers in Severson *et al*. [9], reveals that 26 of the 146 single-copy genetic markers used are present more than once in the genome assembly (data not shown). The high percentage of repeated single-copy markers from a well-known study presents the possibility that these duplicated assembly regions may have resulted from actual segmental duplications, haplotype polymorphisms or misassemblies.

If these regions represented segmental duplications, they would have to be physically close to each other - as the genetic markers have been extensively used and the genetic positions calculated have been well characterized and fall out as one genetic locus [9]. However, the genome assembly has these repeated single-copy markers sometimes localizing to different supercontigs (suggesting a greater distance between them). These different supercontigs sometimes also have markers on them that localize to different linkage groups. This suggests that even though there may be a number of repeated markers present close to each other, a certain degree of misassembly would explain how a single-copy genetic marker would be duplicated on another supercontig or present along with a genetic marker from another linkage group. These events can be explained by the high repeat content of this genome and the presence of repeats flanking these regions, further complicating their proper assembly. It was interesting to note that shotgun sequences from identical repeats were some of the only discrepancies in our assemblies in this study. However, the relatively small size of these assemblies enabled us to completely assemble the BACs correctly.

If these regions represent haplotype polymorphic regions, they should demonstrate genetic drift and therefore a certain amount of sequence variation. These differences would result in the haplotype regions assembling into two scaffolds and therefore complicating the assembly. This phenomenon is seen in polymorphic regions of the *A. gambiae *genome (demonstrating 95-99% similarity) that assembled independently of each other ([4,8] and R. Bruggner and M. Hammond, personal communication). Strains used for sequencing are usually inbred to eliminate usual genomic variation to enable an easier assembly and analysis (the strain of *Ae. aegypti *used for genome sequencing (LVP^ib12^) was inbred for 12 generations from an already inbred strain). However, this cannot eliminate the presence of balanced polymorphisms where homozygous regions result in lethality - a phenomenon extensively used in *Drosophila *genetics. Haplotype polymorphic regions are expected in genome assemblies; however, their negative effects on assembly and analysis can be minimized by proper strain selection and inbreeding. The replicated regions seen here were not precise duplications, as a comparison of the entire nucleotide sequence revealed intergenic differences between the replicated blocks. A comparative analysis revealed that 23 of the 28 transcripts encompassed by these 'replicated' BACs were single copy in both the *A. gambiae *and *D. melanogaster *genomes, again suggesting a single-copy nature. The variation seen between replicated regions, the 'hybrid' nature seen between the BAC sequence and the genomic replicates, the characterization of the markers and encompassed genes as being single copy in *Aedes *[9], as well as in *Anopheles *and *Drosophila*, lead us to believe that these replicated regions in the genome assembly represent polymorphic haplotypes coupled with some misassembly resulting from flanking repeat sequence. There remains the possibility that some of these regions are actually duplicated in the genome and are present close to each other.

The replication of an unusually high percentage of genomic blocks experimentally shown to contain single-copy sequences (57% (8 of 14)), indicates the presence of an assembly issue which affects the number of gene predictions in the gene build and the relation of various scaffolds to each other. This phenomenon also emphasizes the importance of strain selection and proper inbreeding to enable an easier genome assembly. The proper characterization of these probable haplotype regions would enable a better genome assembly and mapping of scaffolds to linkage groups.

### Similarity to *Drosophila *and *Anopheles*

All manually annotated transcripts were compared to the *Drosophila *and *Anopheles *gene sets (see Table 3). Only one annotation (number 19) did not show homology to *Anopheles *or *Drosophila *proteins with the search parameters used. This transcript did demonstrate similarity to an *Aedes *salivary protein (D7cclu23-like salivary protein). When the search parameters were relaxed, the primary hit to *Anopheles *is an odorant-binding protein (OBP49). A salivary- or odorant-related gene would be expected to have significantly diverged from *Anopheles *and even further diverged from *Drosophila *homologs and would not show a high degree or any similarity in the stringent comparative searches used.

Of the remaining 50 transcripts, 50 and 43 demonstrated similarity to the *Anopheles *and *Drosophila *gene sets, respectively. Seven manual annotations that did not have any similarity to the *Drosophila *genome (but did to the *Anopheles *genome) may have either been lost in the lineage that gave rise to the higher dipterans or have significantly diverged from their homologs. Most transcripts had a one-to-one relationship with a gene in the other dipteran genomes. In general, most manually annotated transcripts were similar in length and amino-acid identity to the other dipteran transcripts. The transcripts that had similarity to *Anopheles *transcripts had an average of 72% identity to the manually annotated transcripts with a range 32-100% identity. The 49 *Drosophila *transcripts had an average of around 60% identity with a range 31-97%.

The 51 transcripts represent a gene density of one gene every 30.8 kb, which is considerably lower than in *A. gambiae *[7] or *D. melanogaster *[6]. *Aedes *genes possessed larger intergenic sequences, resulting in multi-exon genes being about four times as large as their *Anopheles *counterparts. This lower gene density seen in *Aedes *can be related to its larger genome size, with a much higher repeat/transposon content.

### Synteny

To look for syntenic relationships, the 11 blocks of transcripts (those with more than one annotation) were compared with the *Anopheles *and *Drosophila *genome (see Table 3). Ten of the blocks had transcripts in them that were similarly clustered in the *Anopheles *genome, whereas only one cluster of two adjacent transcripts was found in *Drosophila*. Overall, 38 transcripts in 10 blocks demonstrated the closer relationship and shorter divergence times between the two mosquitoes [18].

Syntenic studies between genomes can have important applications, including the verification of transcripts and gene annotations. Transcript 38 did not have similarity to any anopheline annotation but possessed significant similarity to a *Drosophila *transcript. This BAC sequence demonstrated synteny to the *Anopheles *genome and was the only transcript missing, although the nucleotide sequence corresponding to this sequence was present. Further investigation revealed that the transcript corresponding to it was removed in the last *Anopheles *gene build. The presence of this transcript in both the *Drosophila *and *Anopheles *genome, as well as the corresponding nucleotide sequence in the *Anopheles *genome, suggests that this anopheline transcript needs to be reinstated.

## Conclusion

This study has resulted in the description of the repeat content, gene content and relationship to other dipteran genomes of 14 *Ae. aegypti *BACs. The high repeat content of this genome adversely affected the assembly and complicated *in silico *annotation. The verification of the haplotype nature of some scaffolds will enable an enhanced assembly and mapping of scaffolds to linkage groups. A well-defined set of *Aedes *transcripts (such as those in this study) combined with *Aedes *ESTs, and the demonstrated similarity to the *A. gambiae *and *D. melanogaster *genome are necessary for a high-quality genome annotation. This study allows us to get an overall view of the genome-assembly quality of this important disease vector and presents a benchmark set of manually annotated and validated transcripts in addition to validating the whole genome auto-annotation.

## Materials and methods

The *Ae. aegypti *BAC library [19] was screened with primers specific to known single-locus genetic markers [9] to isolate BAC clones for further analysis. Fourteen BAC clones were shotgun sequenced and assembled using both the TIGR assembler [2] and Seqman [10]. All shotgun sequences were assembled together to ensure that they sorted independently into the contigs corresponding to individual BAC clones. Primers were designed to single-copy sequence flanking gaps in an effort to close them through PCR and sequencing of the BAC clone. Assembled sequences were analyzed for repetitive/transposon content using Repeatmasker [16] and CENSOR [17], using the arthropod, *D. melanogaster *and *A. gambiae *repeat datasets.

GENESCAN 1.0 [12] and FGENESH 1.0 [13] were used with default parameters and the vertebrate, *D. melanogaster *and *A. gambiae *training datasets to predict genes *in silico*. To identify expressed transcripts, BLAST was performed with these assembled scaffolds to the *Ae. aegypti*, *D. melanogaster *and *A. gambiae *EST databases [2,4,20]. The cutoff used to limit the number of ESTs was e < -100 for *Ae. aegypti *and e < -50 for *Anopheles *and *Drosophila *ESTs.

Evidence used for the manual annotation of the BAC sequences included *ab initio *gene prediction and ESTs from the *Aedes*, *Anopheles *and *Drosophila *genomes. Predicted exons and ESTs that were similar to transposons were not used. BLAST to the *Drosophila *and *Anopheles *peptide datasets was used to capture exons and genes that were not included in *Aedes *ESTs or predicted gene models. Manual annotation was performed using Apollo [14]. Predictions were made conservatively with evidence needed from non-transposon-related *Aedes *ESTs and/or similarity to other dipteran genes/ESTs.

PCR primers were designed to manually annotated transcripts for PCR and sequencing validation. Total RNA was isolated with Trizol Reagent (Invitrogen, Carlsbad, CA) from *Ae. aegypti *LVP^ib12 ^mosquitoes (one to three instar larvae (11.5%); fourth instar larvae and pupae (11.5%); 1-2-day adults (22%); 5-7-day adults (15%); 2-day post-bloodfed female (41%)). cDNA was prepared from the above RNA using the SuperScriptII (Invitrogen) system. Primers were designed across introns when possible. Control primers were designed to the *Ae. aegypti *gene for ribosomal protein 17A (AY064121). PCR was conducted with Platinum *Taq *using 35 cycles. PCR products were gel-purified (QIAquick Gel Extraction Kit, Qiagen, Valencia, CA) and sequenced using primers from the PCR reactions. Sequencing was performed on ABI3730XL (Applied Biosystems, Foster City, CA). Sequence obtained was used to confirm or correct manual annotations and splice sites. Manually annotated transcripts were compared to the *Anopheles *and *Drosophila *genomes (BLASTX, BLOSUM90 [4]) for evaluation of similarity and synteny.

## Additional data files

The following additional data are available with the online version of this paper. Additional data file 1 contains detailed descriptions of the assembly and annotation of each BAC clone, the presence of replicated regions, and orthologous and syntenic relationships.

## Supplementary Material

Additional data file 1 Detailed descriptions of the assembly and annotation of each BAC clone, the presence of replicated regions, and orthologous and syntenic relationships. Click here for file
