# Supplementary material for: Analysis of 14 BAC sequences from the Aedes aegypti genome: a benchmark for genome annotation and assembly
Source: Genome Biol. 2007 May 22;8(5):R88. doi: 10.1186/gb-2007-8-5-r88 (PMC1929151; doi:10.1186/gb-2007-8-5-r88)
Supplement: Additional data file 1 — Detailed descriptions of the assembly and annotation of each BAC clone, the presence of replicated regions, and orthologous and syntenic relationships. [file gb-2007-8-5-r88-S1.doc]

Summary of each BAC clone

BAC1 (ND13I3; GenBank: EF173370) contained the *Ae. aegypti* ribosomal protein *L17A* (*RpL17A*; GenBank: AY064121) which was genetically mapped to chromosome 2q [9].

This BAC clone was assembled into two oriented contigs totaling 82203 bp. Two of the three gaps were closed with primers designed to single copy sequence flanking the gaps. Primers designed to the remaining three gaps did not produce readable sequence. Manual annotations resulted in six gene models (1-6). All gene models had identical nucleotide matches in the *Aedes* genome and included the *RpL17A* gene. A comparison to the *Anophleles* genome revealed that all six *Aedes* gene models were clustered in the *Anophleles* genome on chromosome 3R. All six orthologues were found in the *D. melanogaster*  genome, though they were not clustered. With the exception of one transcript from a multigene family, all transcripts from the *Drosophila* and *Anophleles* genome were orthologues of each other.

On comparison to the *Aedes* genome assembly, all six transcripts were found on Supercont1.789. However, three transcripts were also found on Supercont1.1137 while the other three were also found on Supercont1.1393 and Supercont1.875. Transcripts present more than once were virtually identical in sequence and intron-exon structure. To see if this represented either duplicated, misassembled or haplotype polymorphic regions, the whole genomic region (including introns) encompassing each of the six transcripts was compared to corresponding regions on the scaffolds in the genome assembly. Supercont1.789, which had all six transcripts, only the first three (1-3) had identical intergenic sequence, while their duplicated counterparts had intergenic sequence that varied slightly. Of the other three transcripts (4-6), Transcript 4 matched a transcript on Supercont1.875. Interestingly, the 3' region of this gene matched corresponding areal of a transcript on Supercont1.1393, and the 5' region lined up to the corresponding area of transcript on Supercont1.789. Intergenic sequence was only identical to that on Supercont1.875. The sequence on the two other Supercontigs (1.789 and 1.393) had an addition gene annotation on them that was not present on this BAC sequence or on Supercont1.875. The *Rpl17A* gene (Transcript 5) was present three times in the genome. The transcript found on Supercont1.393 was identical to that found on the BAC sequence while the other two transcripts varied in intergenic sequence. The last transcript (6) represents the 5' exon and part of the first intron of a transcript. It is found on two Supercontigs and none of the intergenic sequences were the same as that found on the BAC clone. This BAC represents the most complicated assembly structure in the genome that corresponded to a single BAC clone.

BAC2 (ND22N19; GenBank: EF173371) has the genetic marker *D6L600* (GenBank: BH214535) and was genetically mapped to chromosome 2q [9]. This BAC clone was assembled into 2 oriented contigs. Four gaps were closed with primer walking. The resulting two scaffolds totaled 146563 bp. Though *in silico* annotation produced several gene models, manual annotation of this region resulted in no transcripts. All putative ORFs were found to be associated with transposons. This BAC had the highest G+C content (47.09%). Identical sequence was found in on Supercont1.14 (BlastN). However, similar sequence encompassing this whole BAC was also found on Supercont1.141, Supercont1.154, Supercont1.153 and Supercont1.298.

BAC3 (ND22N5; GenBank: EF173372) contained the Maltase precursor (*Mal1*; GenBank: M30442) which was genetically mapped to chromosome 3p [9]. This BAC clone was assembled into 1 scaffold after 3 gaps were closed with primers. The 2 contigs totaled 116923 bp. Manual annotations resulted in only 1 gene model – Mal1 (7). This gene had multiple hits in both the *Anophleles* and *Drosophila* genome though the primary hit in each other Dipteranl genome was the orthologue of each other. This transcript was found on two Supercontigs (1.7 and 1.403). Though the single exon transcript was identical on both supercontigs, non-coding sequence was identical only on Supercont1.403.

BAC4 (ND41B18; GenBank: EF173373) contained the genetic marker *LF347* (GenBank: T58329) which mapped to 3p [9]. This BAC clone was assembled into one scaffold(164547 bp). Manual annotations resulted in six gene models (8-13). All six gene models had matches in the *Anophleles* and *Drosophila* genomes and the Dipteran genes demonstrating the most similarity were orthologues of each other. These were all present on Supercont1.301. Though manually annotated gene model 9 had a slightly different intron-exon structure from the *Aedes* match, it had matches in both the *Anophleles* and *Drosophila* genome. This gene structure was confirmed with RT-PCR and sequencing. The putative orthologue in each Dipteran genome was the orthologue of the other. Transcript 8 belonged to a multi-gene family and was present in an intron (on the opposite strand) of transcript 9. Manually annotated transcript 10 was longer than the genome annotated transcript with its 5' end extended to the start codon. Both transcripts 12 and 13 were identical to transcripts on supercont1.301. When comparing these transcripts to the *Anopheles* genome to look for syntenic relationships, three putative *Anopheline* orthologues corresponding to transcripts 9,11 and 12 were clustered on *Anophleles* 2R.

BAC5 (ND41C6; GenBank: EF173374) contained the vitelline membrane protein homologue (*15a*; GenBank: AAU91682) gene which mapped to 3p [9]. This BAC clone was assembled into seven contigs totaling 89409 bp. Manual annotations resulted in two gene models (14 and 15). Both gene models had matches in the *Anophleles* but not the *Drosophila* genome. These may represent genes that have been lost in the higher dipteran lineage or genes specific to mosquitoes. Transcript 15 matched two nested genes in the *Anophleles* database. Based on EST evidence, these may have been obtained from splitting a single gene which would be the orthologue of the *Aedes* transcript. Both gene models were present with identical exons on *Aedes* Supercontig1.116. Only 15 was present on Supercontig1.216, while 14 was not annotated. The genome sequence corresponding to this transcript on Supercont1.216 was identified as being of a repeat nature and may have been masked during annotation. The transcript, if annotated did possess a number of amino acids in the coding sequence that differed from the BAC transcript as well as that on Supercont1.116.

BAC6 (ND46O19; GenBank: EF173375) contained genetic marker *BA67* (GenBank: AI561370) which mapped to 2q [9]. This BAC clone was assembled into seven contigs totaling 114988 bp. Manual annotations resulted in three gene models (16-18) all of which had orthologues in *Anophleles* and *Drosophila*. All three were present in a cluster on 3R in the *Anophleles* genome. The three gene models were present twice in the genome, on Supercont1.1232 and Supercont1.1132. A comparison of the duplicated transcripts revealed differences in intergenic sequence. Intergenic sequence was identical to that on Supercontigs1.1232. Gene model 16 had differently manually annotated 3' terminal exons based on EST evidence.

BAC7 (ND48J19; GenBank: EF173376) contained genetic marker *D7* (GenBank: MQSD7AB) which mapped to 2q [9]. This BAC clone was assembled into one contig totaling 83496 bp with four manual annotations (19-21). Gene mode 19 was the most divergent and the stringent parameters had to be relaxed to find homology in *Anophleles*. Gene models 21 and 22 were similar to each other and both represent the short form of the *D7cclu23*-like salivary gland protein which is also found in tandem in *Anophleles*. Gene model 20 has high homology to *histone H3* but is not annotated in this region of the *Aedes* genome.

BAC8 (ND56P6; GenBank: EF173377) contained the sodium channel protein *para* (Protein paralytic; GenBank: AF468968). para was genetically mapped to 3q [9]. This BAC clone was assembled into a single 81099 bp sequence. There was a single manually annotated gene model (23) on this BAC corresponding to the first seven exons of *para*. This gene encompasses an area larger than this BAC as evidenced by EST and comparative data. Though this gene belongs to a multi-gene family, the gene with the highest similarity to it in *Anophleles* was the orthologue of the hit in *Drosophila*. This sequence had a high degree of similarity in two supercontigs in the *Aedes* genome – Supercont1.312 and Supercont1.816. Though coding sequence was identical in both cases, the intron sequence on Supercont1.312 differed from that on this BAC.

BAC9 (ND67B23; GenBank:EF173378) contained the genetic marker *LF106* (GenBank: BM005490) which was genetically mapped to 3q [9]. This BAC clone was assembled into two contigs totaling 136645 bp with six manually annotated gene models (24-29). Gene models had orthologues in the *Drosophila* and *Anophleles* genome, both of which were orthologues of each other. Four transcripts were present in a cluster in the *Anophleles* genome on chromosome 2R. These transcripts were present twice in the *Aedes* genome on Supercont1.1 and Supercont1.488. Though coding regions were very similar, intergenic sequence for five transcripts was identical on Supercont1.488 while the last transcript (28) had intergenic sequence identical to that on Supercont1.1.

BAC10 (ND-83_P15; GenBank: EF173379) contained the genetic marker *AEG128* (GenBank: BI096849) which was mapped to 3p [9]. This BAC clone was assembled into two contigs totaling 76584 bp with three manually annotated gene models (30-32). All gene models were found once in the *Aedes* genome assembly on Supercont1.288. Transcript 30 matched the *Spätzle 4* (*Spz4*) protein in *Anophleles* and *Drosophila*. The *Aedes* genome annotation of this gene is incomplete and corresponded to only the 5' area of the gene.

BAC11 (105H24; GenBank: EF173366) contained the cDNA genetic marker *LF178* (GenBank: T58309) which was mapped to 1p [9]. This BAC clone was assembled into two contigs totaling 140290 bp with four manually annotated gene models (33-36). All four manually annotated gene models were found in the *Anophleles* and *Drosophila* genomes. *Anophleles* and *Drosophila* transcripts were orthologues of each other. Three of the four transcripts were found in a cluster in *Anophleles* on chromosome X, while two of the transcripts were found flanking each other in *Drosophila* on the X chromosome as well. These two represent the only two transcripts that demonstrated some degree of synteny with *Drosophila*. All transcripts had identical matches in the *Aedes* genome assembly on Supercont1.59. Manually annotated transcript 34 lacked an exon (that had no supporting evidence) present in the gene build. This BAC clone encompassed an area of this scaffold that had three novel annotations that had similarity to a Rhabdovirus nucleocapsid protein (suggesting a genomic integration). Though EST evidence suggests they are expressed, they are expressed as multiple transcripts none of which precisely match the annotations or show similarity to other Dipteran genomes. These were not included in the set of manually annotated transcripts.

BAC12 (124C17; GenBank: EF173367) contained the genetic marker *LF138* (GenBank: T58332) which was mapped to 2q [9]. This BAC clone was assembled into eight contigs totaling 158121 bp with six manually annotated gene models (37-42). All manually annotated gene models had hits in the *Anophleles* and *Drosophila* genomes. Gene model 38 hit a region of the *Anophleles* genome missing a transcript (removed in the most recent gene build) suggesting that this needs to be reinstated. This gene is present once in the genome of *Drosophila* and *Aedes* and each is the orthologue of the other. All other *Drosophila* matches were orthologues of the *Anophleles* match. Five of the six gene models were present in a cluster on chromosome 3R in *Anophleles*. All transcripts were found in the *Aedes* assembly on Supercont1.25.

BAC13 (26O21; GenBank: EF173368) contained the genetic marker *LF342* (GenBank: BM005512) which was mapped to 2p [9]. This BAC clone was assembled into two contigs totaling 87550 bp with four manually annotated gene models (43-46).

Three of the annotations were very similar and hit the same gene family in *Drosophila* as well as *Anophleles*. The fourth annotation was found in *Anophleles*, next to the transcript demonstrating the highest similarity to the other three transcripts. However, this fourth transcript was not found in *Drosophila*.

The four manually annotated transcripts were found in the *Aedes* assembly in two supercontigs – 1.348 and 1.39. A comparison of intergenic sequence revealed that even though coding sequence was virtually identical, intergenic sequence from the BAC was identical to that in Supercont1.348.

BAC14 (92LO9; GenBank: EF173369) contained the genetic marker *LF253* (GenBank: T58331) which was mapped to 3p [9]. This BAC clone was assembled into three contigs totaling 93207 bp with four complete gene models (47-50) and a fifth (51) that consisted of the 5' exon of a gene. All five transcripts were found in the *Drosophila* and *Anophleles* genome. They were found in a cluster in *Anophleles* on 2R. The transcript demonstrating homology to transcript 50 was present three times in tandem in the *Anophleles* genome. The four complete gene models were found twice in the *Aedes* genome on Supercont1.140 and Supercont1.146. Though these duplicated gene models had almost identical coding sequence, only the first two (47 and 48) were identical (coding and intergenic) to the corresponding region on Supercont1.140 while the remaining three, including the partial 5' transcript were identical to the corresponding transcripts on Supercont1.146.
